# Supplementary material for: Optimization of Resveratrol Used as a Scaffold to Design Histone Deacetylase (HDAC-1 and HDAC-2) Inhibitors
Source: Pharmaceuticals (Basel). 2022 Oct 13;15(10):1260. doi: 10.3390/ph15101260 (PMC9611521; doi:10.3390/ph15101260)

**Physico-chemical properties**	**Values**											
	RVT	tucidinostat	(4)	(5)	(7)	(20)	(21)	(22)	(23)	(24)	(25)	(26)
Num. heavy atoms	17	29	25	25	24	24	24	25	25	27	35	38
Num. arom. heavy atoms	12	18	18	18	18	18	18	18	18	18	24	24
Fraction Csp^3	0.00	0.05	0.00	0.10	0.00	0.05	0.05	0.05	0.05	0.05	0.04	0.04
Num. rotatable bonds	2	8	5	6	5	6	6	6	6	7	7	8
Num. H bonds receptors	3	4	1	1	1	2	1	3	2	3	4	6
Num. H bonds donors	3	3	3	3	2	2	3	3	3	3	2	2
Log $P_{o/w}$ (iLOGP)	1.71	2.51	2.47	2.50	2.86	2.69	2.58	2.43	2.39	2.12	3.21	2.52
Water solubility (mg/mL) (ESOL)	5.51 e-2	8.93 e-2	1.34 e-2	1.64 e-2	1.26 e-2	1.99 e-2	1.99 e-2	2.92 e-2	4.76 e-2	2.02 e-1	2.17 e-3	2.05 e-3
class	Soluble	Soluble	Moderately soluble	Moderately soluble	Moderately soluble	Moderately soluble	Moderately soluble	Moderately soluble	Soluble	Soluble	Moderately soluble	Moderately soluble
GI absorption	High	High	High	High	High	High	High	High	High	High	High	Low
BBB permeant	Yes	No	No	No	Yes	Yes	Yes	No	No	No	No	No
P-gp substrate	No	Yes	No	No	No	No	No	No	No	No	No	No
CYP1A2 inhibitor	Yes	Yes	Yes	Yes	Yes	Yes	Yes	Yes	Yes	No	No	No
CYP2C19 inhibitor	No	Yes	Yes	Yes	Yes	Yes	Yes	Yes	Yes	No	Yes	Yes
CYP2C9 inhibitor	Yes	Yes	Yes	Yes	Yes	Yes	Yes	Yes	Yes	Yes	Yes	Yes
CYP2D6 inhibitor	No	Yes	Yes	Yes	Yes	Yes	Yes	Yes	Yes	Yes	Yes	No
CYP3A4 inhibitor	Yes	Yes	Yes	Yes	Yes	Yes	Yes	Yes	Yes	Yes	Yes	Yes

Log *kp* (skin permeation)	-5.47 cm/s	-7.05 cm/s	-5.71 cm/s	-5.76 cm/s	-5.53 cm/s	-5.73 cm/s	-5.72 cm/s	-6.07 cm/s	-6.30 cm/s	-7.22 cm/s	-6.23 cm/s	-6.63 cm/s
Lipinski	Yes	Yes	Yes	Yes	Yes	Yes	Yes	Yes	Yes	Yes	Yes	Yes (1 violation: MW>500)
Ghose	Yes	Yes	Yes	Yes	Yes	Yes	Yes	Yes	Yes	Yes	No (1 violation: MR>130)	No (2 violations: MW>480, MR>130)
Veber	Yes	Yes	Yes	Yes	Yes	Yes	Yes	Yes	Yes	Yes	Yes	No (1 violation: TPSA>140)
Egan	Yes	Yes	Yes	Yes	Yes	Yes	Yes	Yes	Yes	Yes	Yes	No (1 violation: TPSA>131.6)
Muegge	Yes	Yes	Yes	Yes	Yes	Yes	Yes	Yes	Yes	Yes	Yes	Yes
Bioavailability score	0.55	0.55	0.55	0.55	0.55	0.55	0.55	0.55	0.55	0.55	0.55	0.55
PAINS alert	0	0	0	1 (anil_no_alk)	0	0	0	0	1 (anil_no_alk)	0	0	0
Synthetic accessibility	2.02	2.84	2.33	2.09	2.25	2.08	1.89	2.16	2.19	2.48	2.85	3.42

Figure S1. IC_{50} curve of compound (**5**) against HDAC-1.

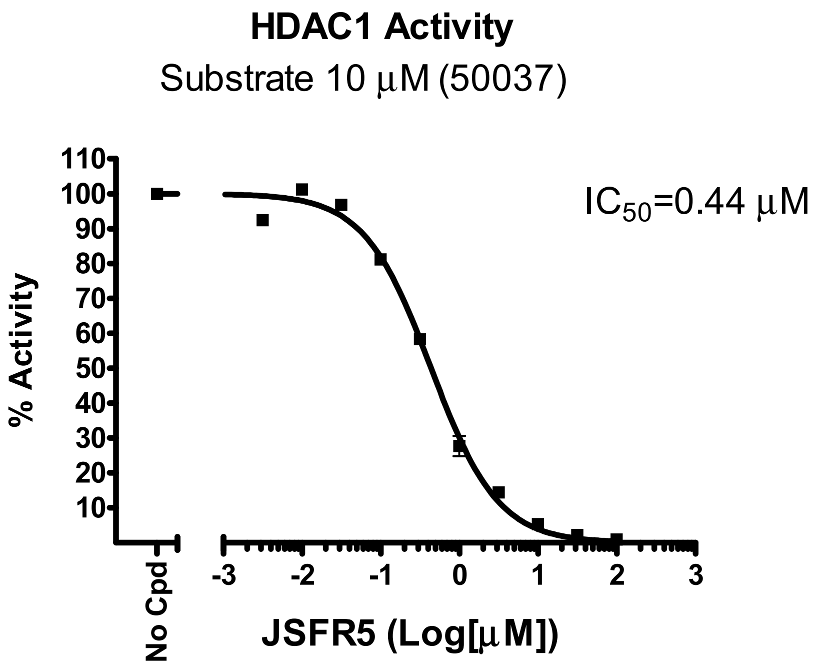

Figure S2. IC_{50} curve of compound (**5**) against HDAC-2

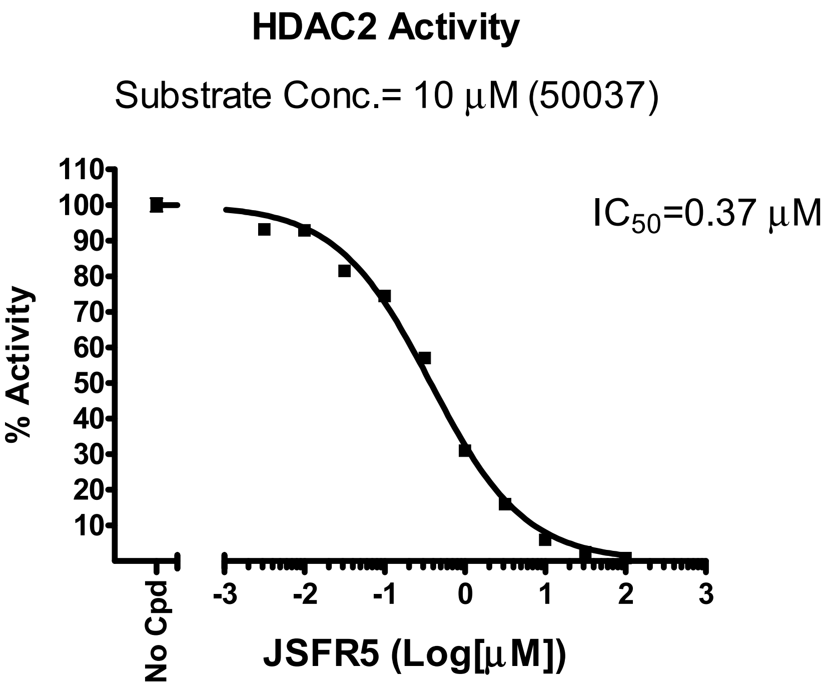

Figure S3. Pose of RVT in the active site of HDAC-1 (map of electrostatic potential – minimum -0.3 and maximum +0.3).

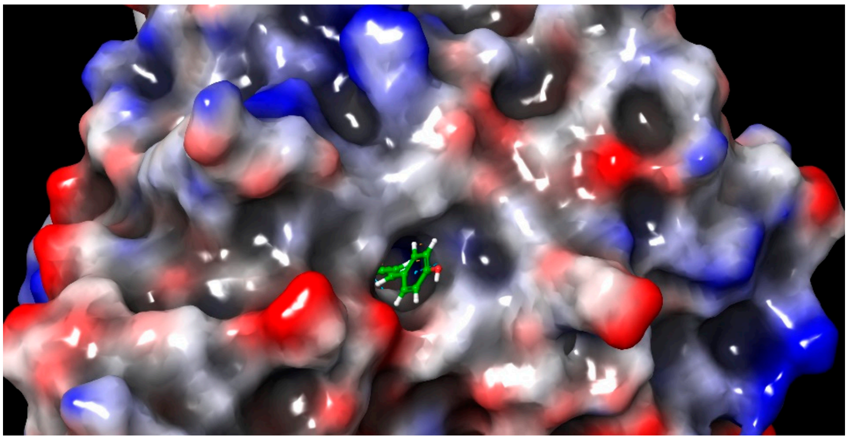

Figure S4. Pose of RVT in the active site of HDAC-2 (map of electrostatic potential – minimum -0.3 and maximum +0.3).

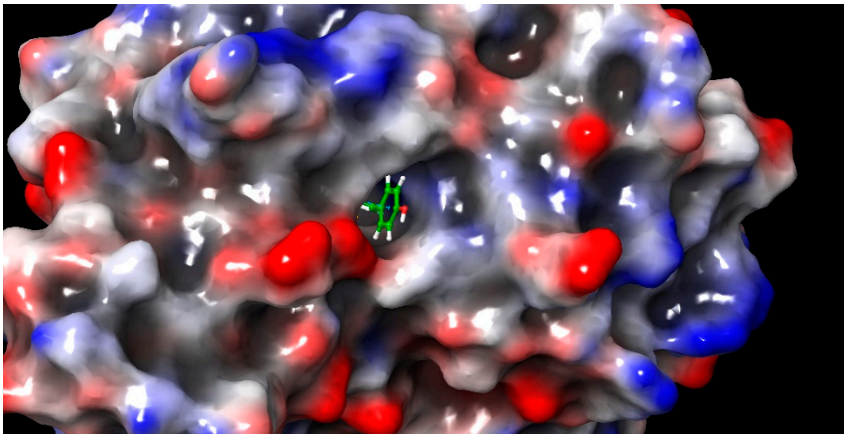

Figure S5. 2D pose of compound (**4**) in the active site of HDAC-2 and its interactions.

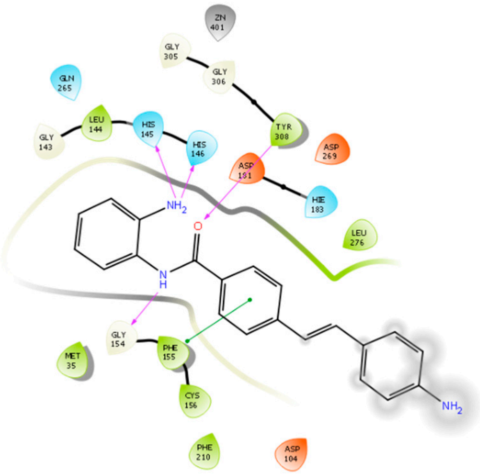

Figure S6. 2D pose of compound (**5**) in the active site of HDAC-2 and its interactions.

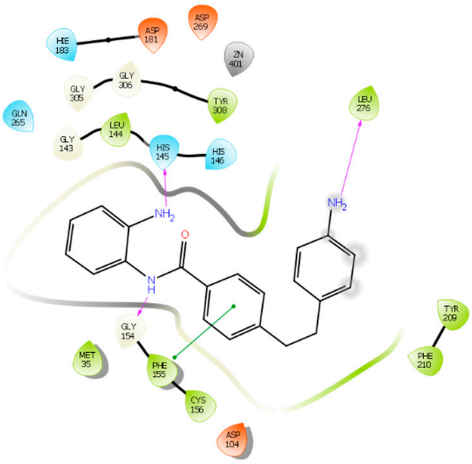

Figure S7. 2D pose of compound (**20**) in the active site of HDAC-2 and its interactions.

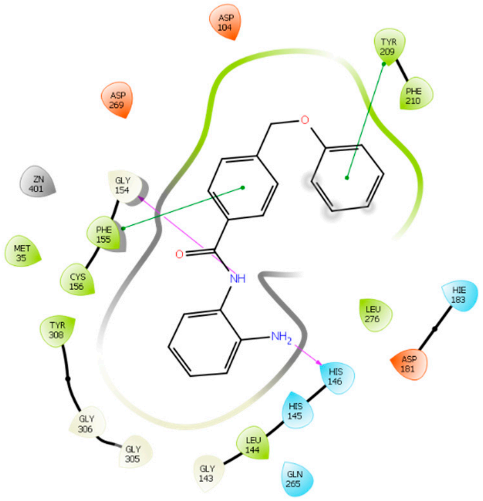

Figure S8. 2D pose of compound (**21**) in the active site of HDAC-2 and its interactions.

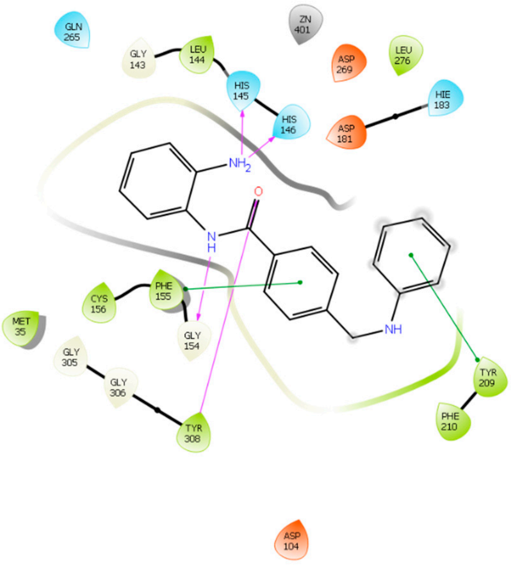

Figure S9. 2D pose of compound (**22**) in the active site of HDAC-2 and its interactions.

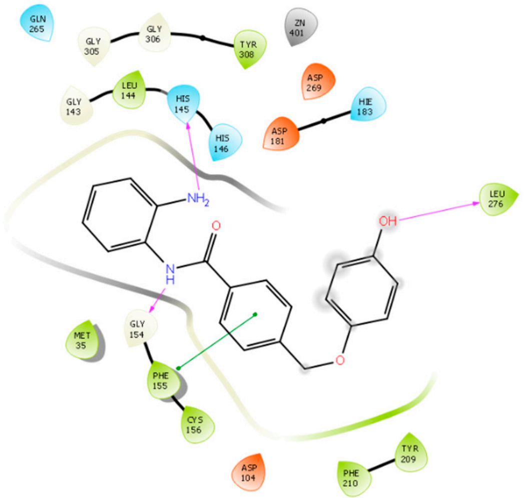

Figure S10. 2D pose of compound (**23**) in the active site of HDAC-2 and its interactions.

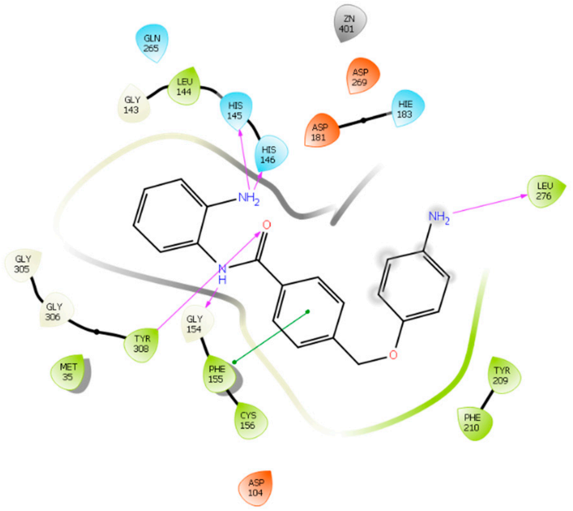

Figure S11. 2D pose of compound (**24**) in the active site of HDAC-2 and its interactions.

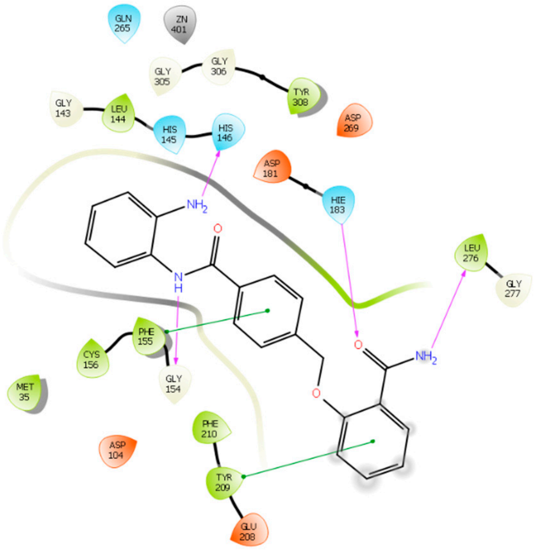

Figure S12. 2D pose of compound (**25**) in the active site of HDAC-2 and its interactions.

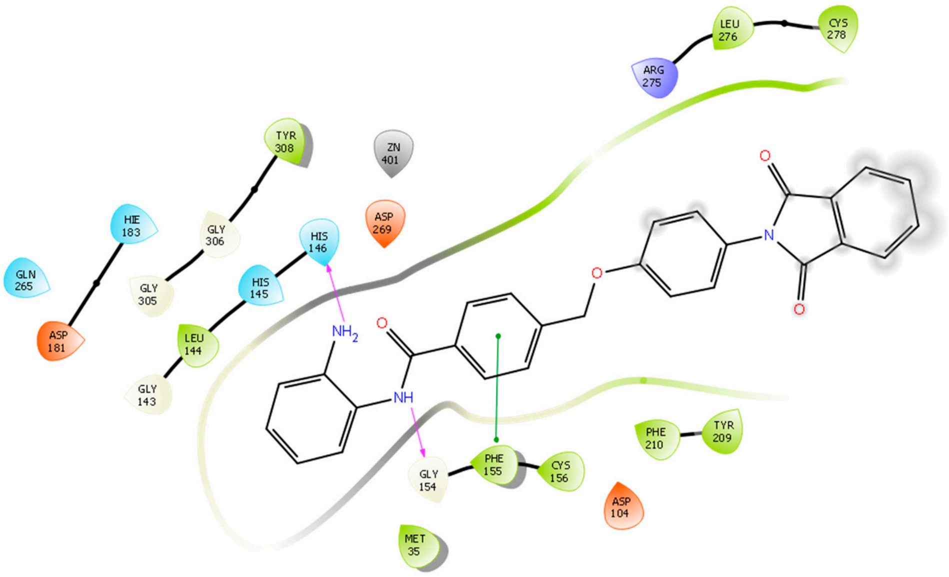

Figure S13. 2D pose of compound (**26**) in the active site of HDAC-2 and its interactions.

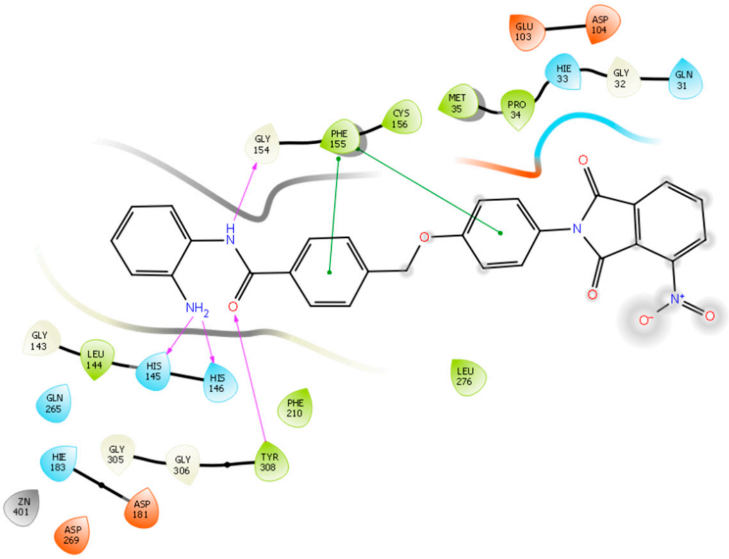

Table S1: Occurrence of hydrogen bonding between protein and ligand, during each replicate of HDAC-1-(**5**), HDAC-2-(**4**), and HDAC-2-(**5**) simulations (HDAC-2 numbering).

	**GLY 154**	**HIS145**	**HIS146**
		**HDAC-1-(5)**	
**Replicate 1**	0.4972	0.8610	0.4544
**Replicate 2**	0.4552	0.8884	0.4820
**Replicate 3**	0.3350	0.8210	0.5100
**Replicate 4**	0.5520	0.8632	0.5556
**Replicate 5**	0.6010	0.8552	0.5934
		**HDAC-2-(5)**	
**Replicate 1**	0.4896	0.7750	0.5244
**Replicate 2**	0.5152	0.7898	0.4584
**Replicate 3**	0.5620	0.8046	0.4426
**Replicate 4**	0.4116	0.8176	0.4358
**Replicate 5**	0.5122	0.8052	0.5332
		**HDAC2-(4)**	
**Replicate 1**	0.4994	0.7882	0.5008
**Replicate 2**	0.4170	0.8168	0.5214
**Replicate 3**	0.4364	0.7790	0.5044
**Replicate 4**	0.4902	0.7562	0.4904
**Replicate 5**	0.4442	0.7878	0.4752

Figure S14: RMSD of protein for each replicate of HDAC-1-(**5**) simulation.

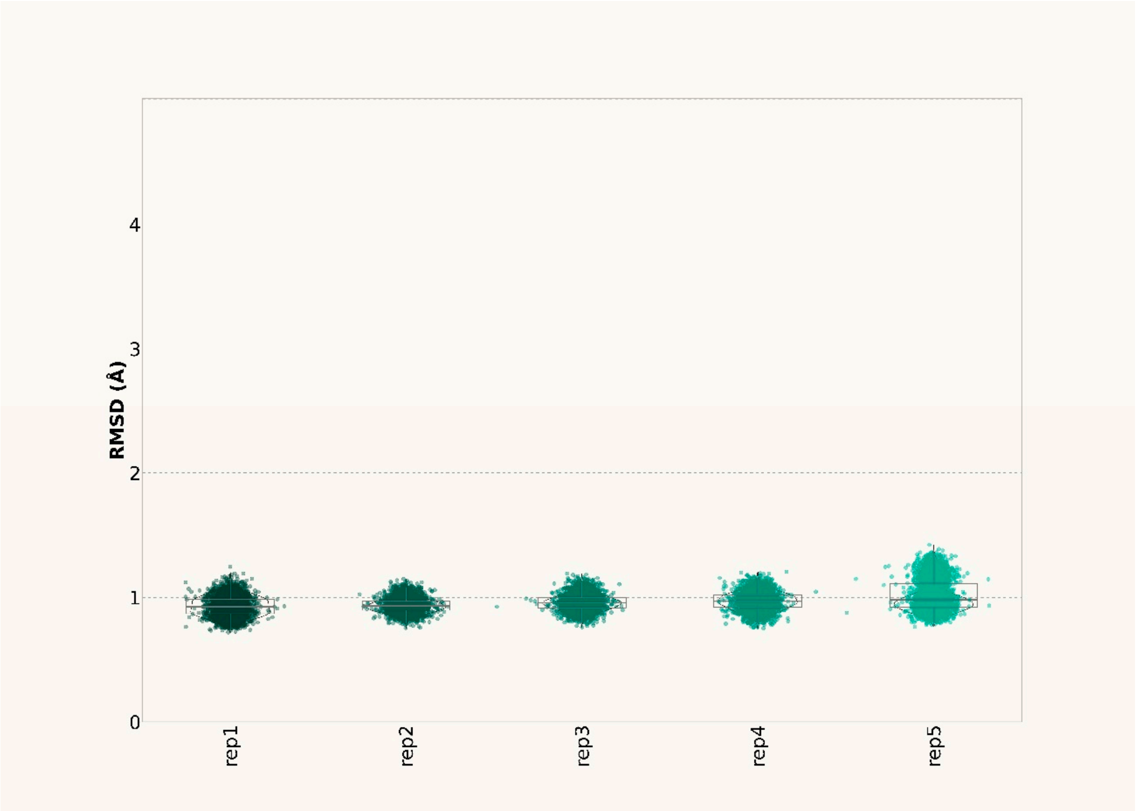

Figure S15: RMSD of protein for each replicate of HDAC-2-(**4**) simulation.

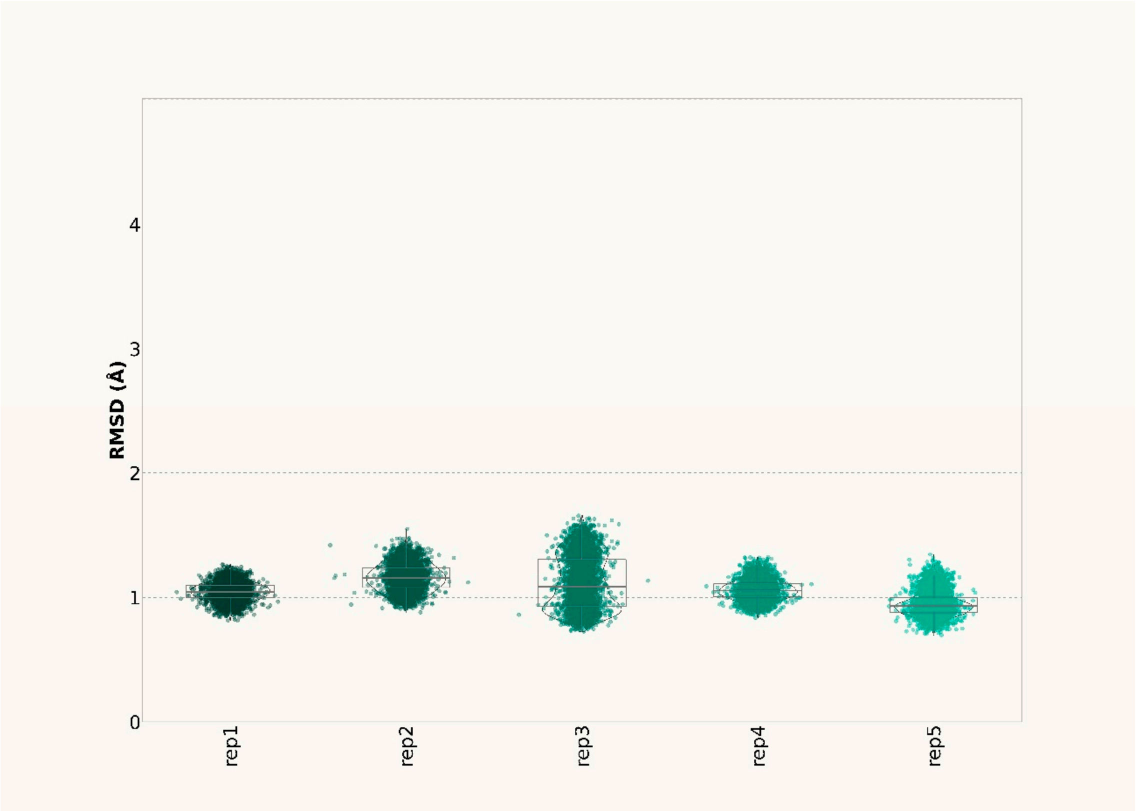

Figure S16: RMSD of protein for each replicate of HDAC-2-(**5**) simulation.

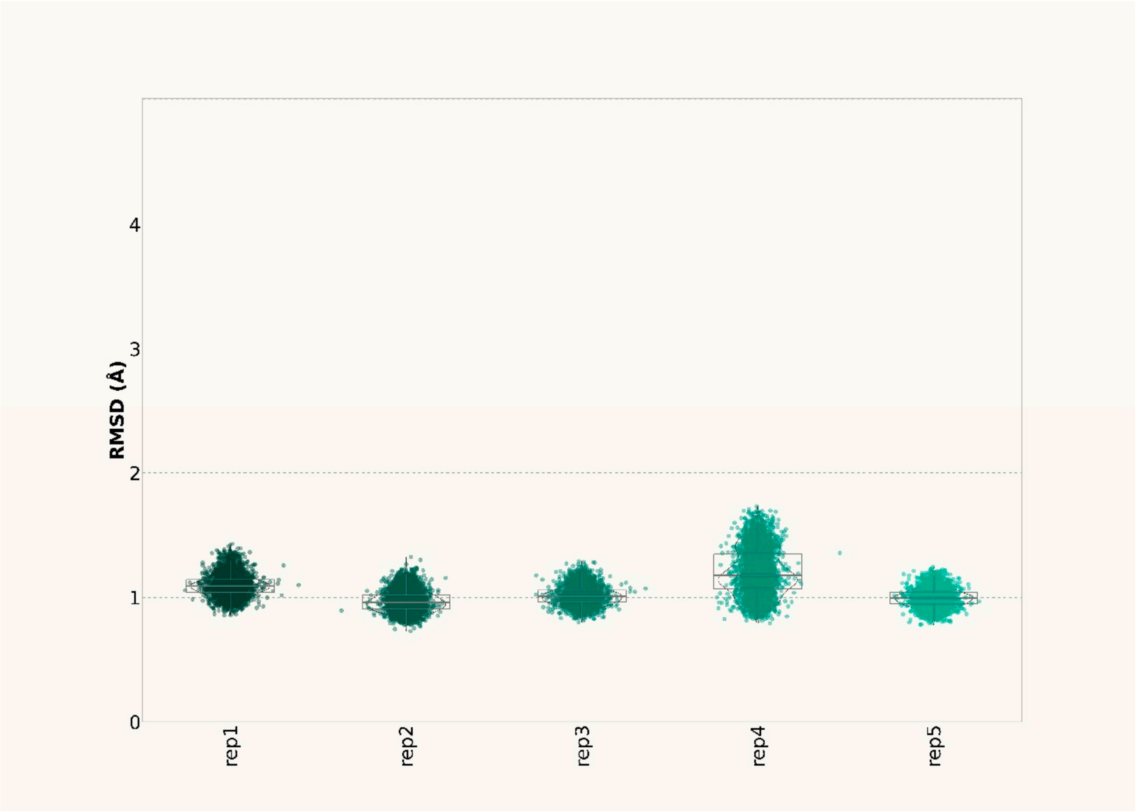

Figure S17: RMSD of heavy atoms of compound (**4**) during each replicate of HDAC-2-(**4**) simulation, compared with docking pose.

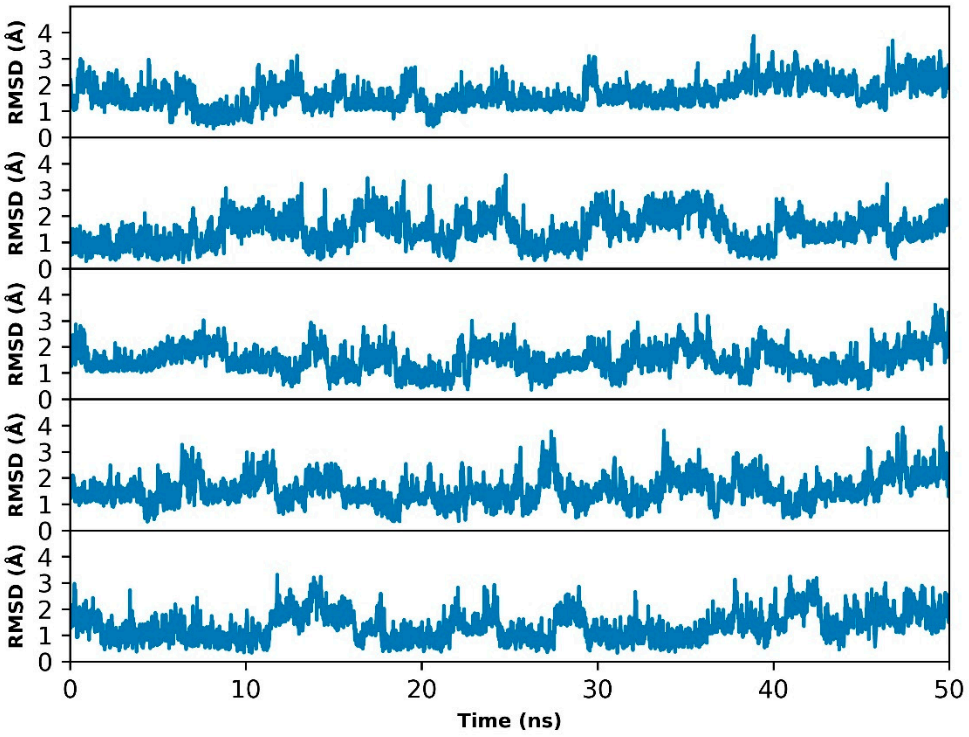

Figure S18: RMSD of heavy atoms of compound (**5**) during each replicate of HDAC-1-(**5**) simulation, compared with docking pose.

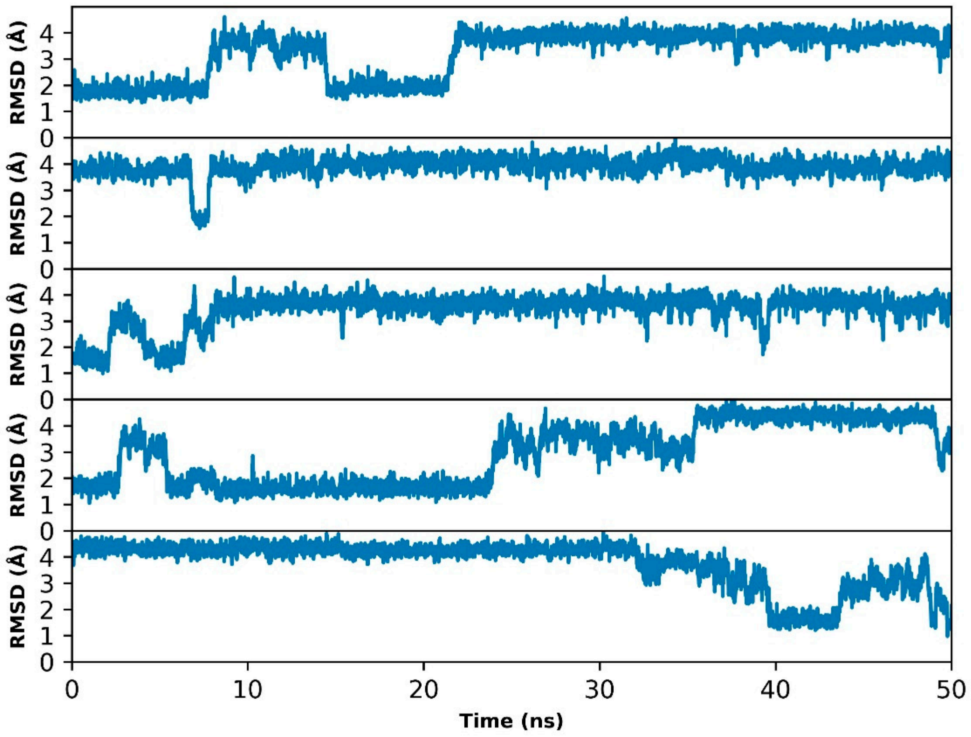

Figure S19: RMSD of heavy atoms of compound (**5**) during each replicate of HDAC-2-(**5**) simulation, compared with docking pose.

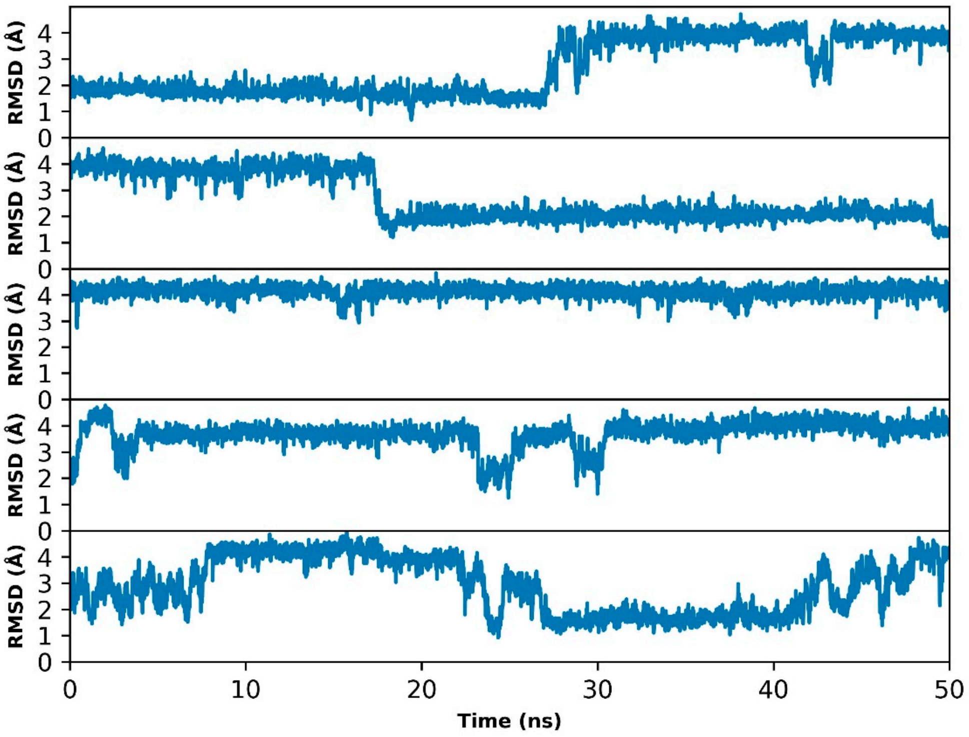

Figure S20: Distance between center of mass of 4-aminophenyl ring to the hydrogen atom of PHE155 for each replicate of HDAC-1-(**5**) simulation.

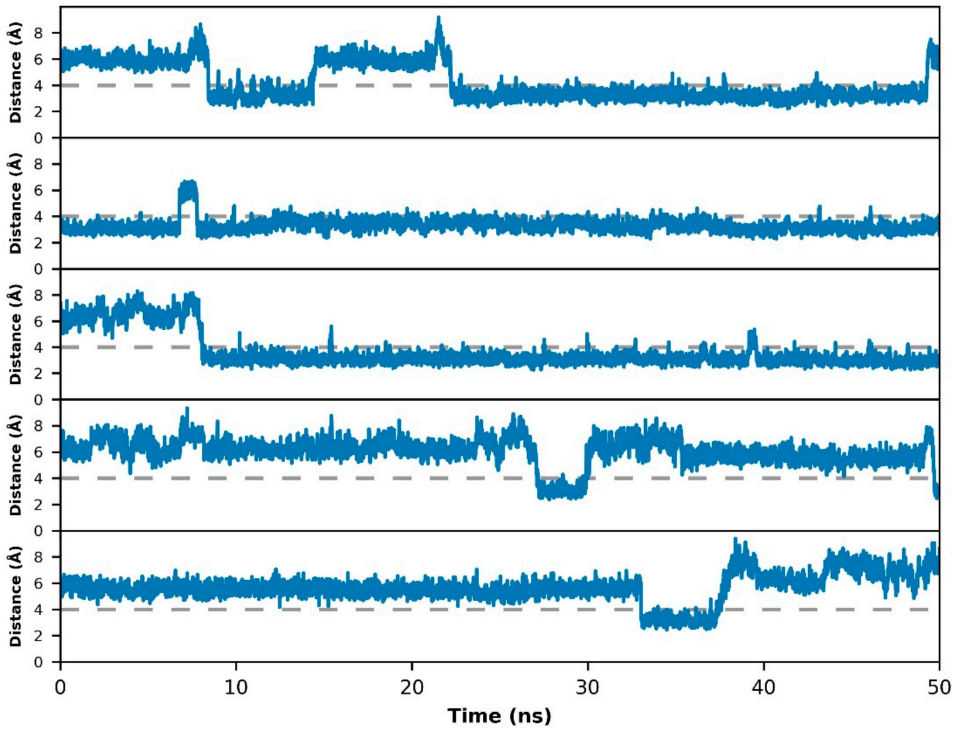

Figure S21: Distance between center of mass of 4-aminophenyl ring to the hydrogen atom of PHE155 for each replicate of HDAC-2-(**5**) simulation.

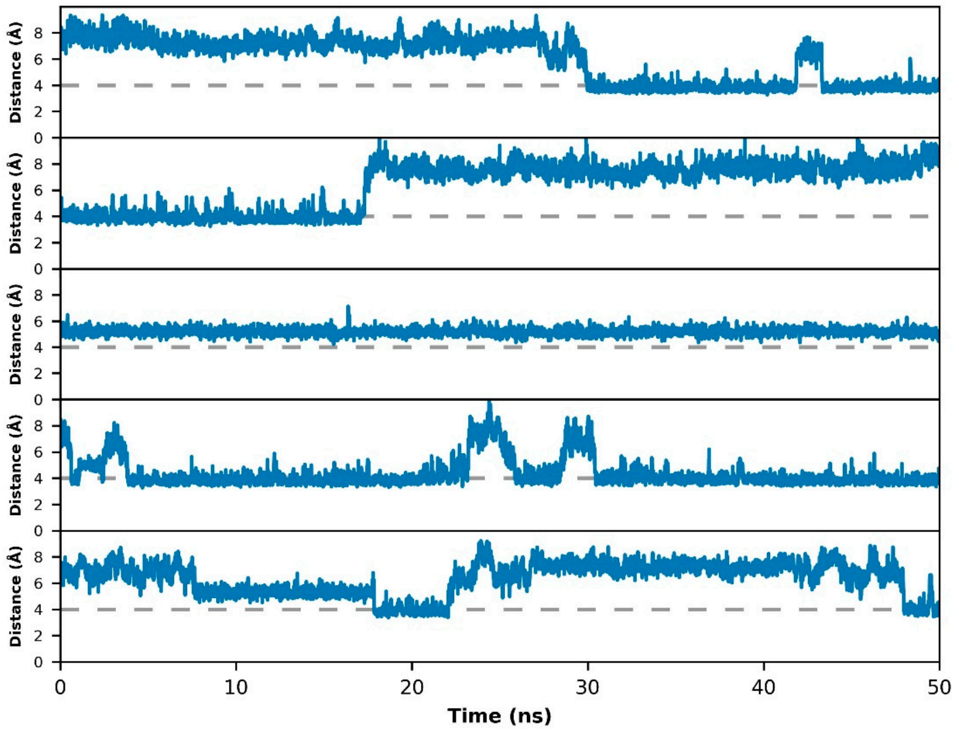

Figure S22: ^{1}H NMR spectra of compound (**4**) (600MHz, DMSO*d6).*

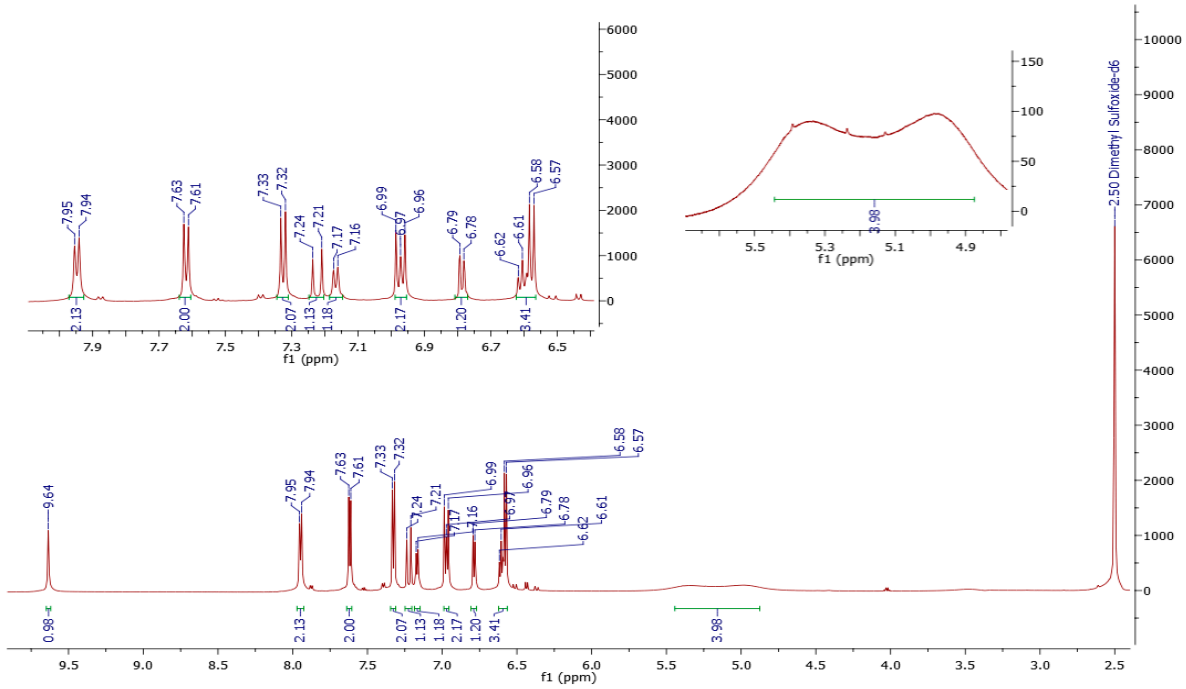

Figure S23: ^{13}C NMR spectra of compound (**4**) (150MHz, DMSO*d6).*

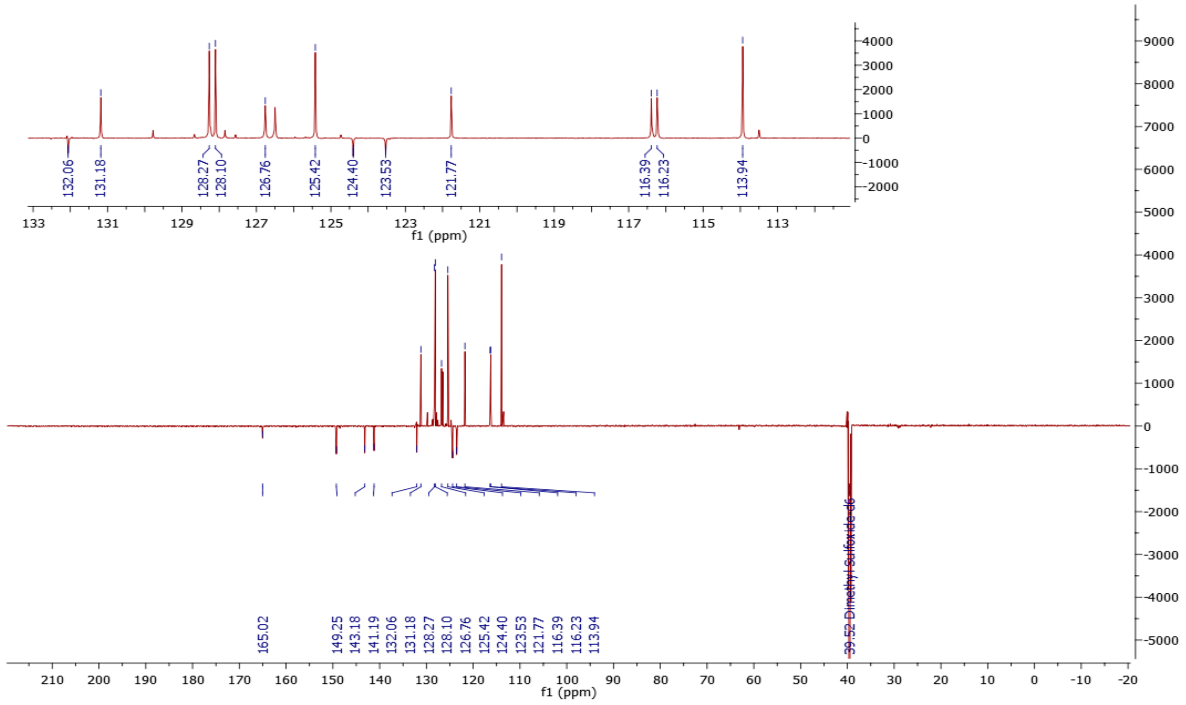

Figure S24: ^{1}H NMR spectra of compound (**5**) (600MHz, DMSO*d6).*

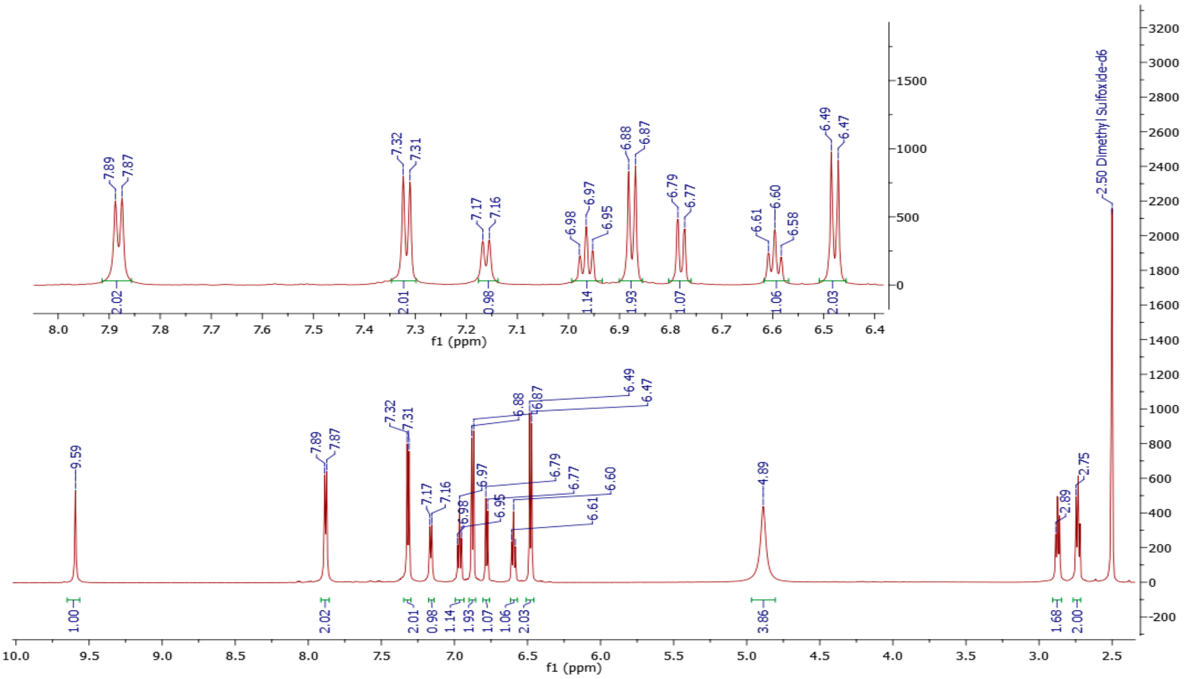

Figure S25: ^{13}C NMR spectra of compound (**5**) (150MHz, DMSO*d6).*

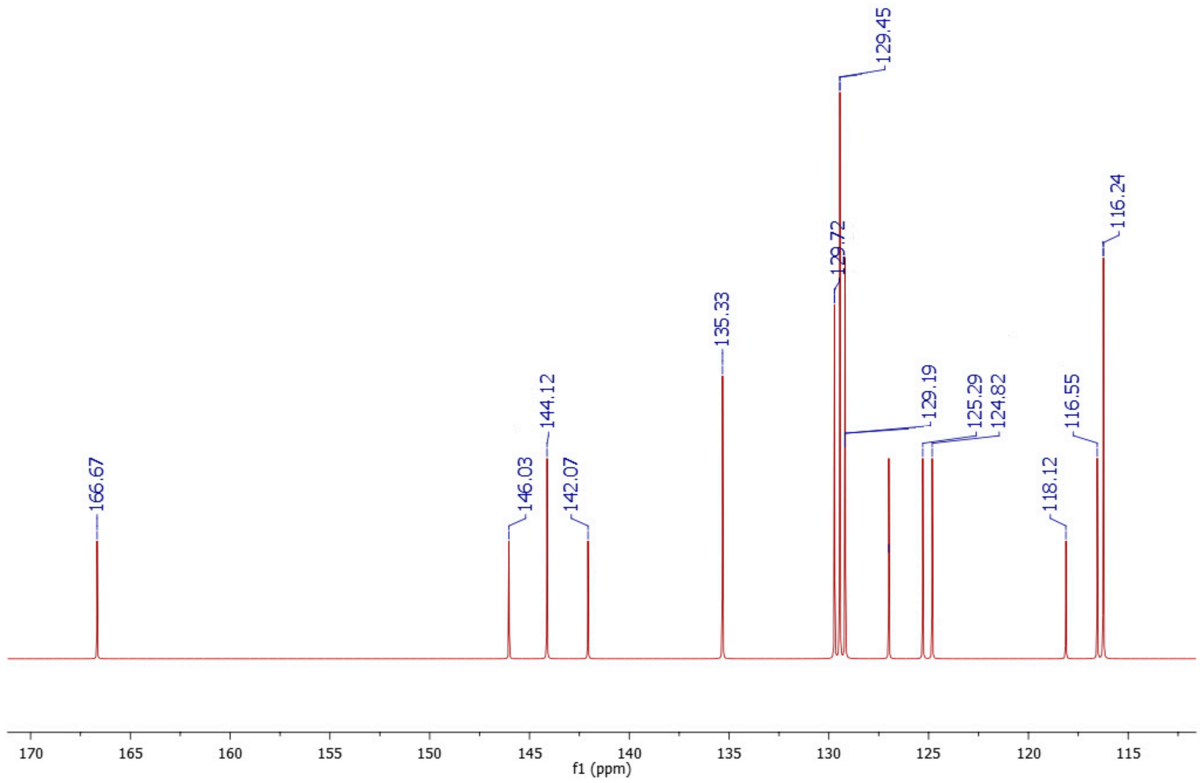

Figure S26: ^{1}H NMR spectra of compound (**7**) (600MHz, $(CD_3)_2CO$).

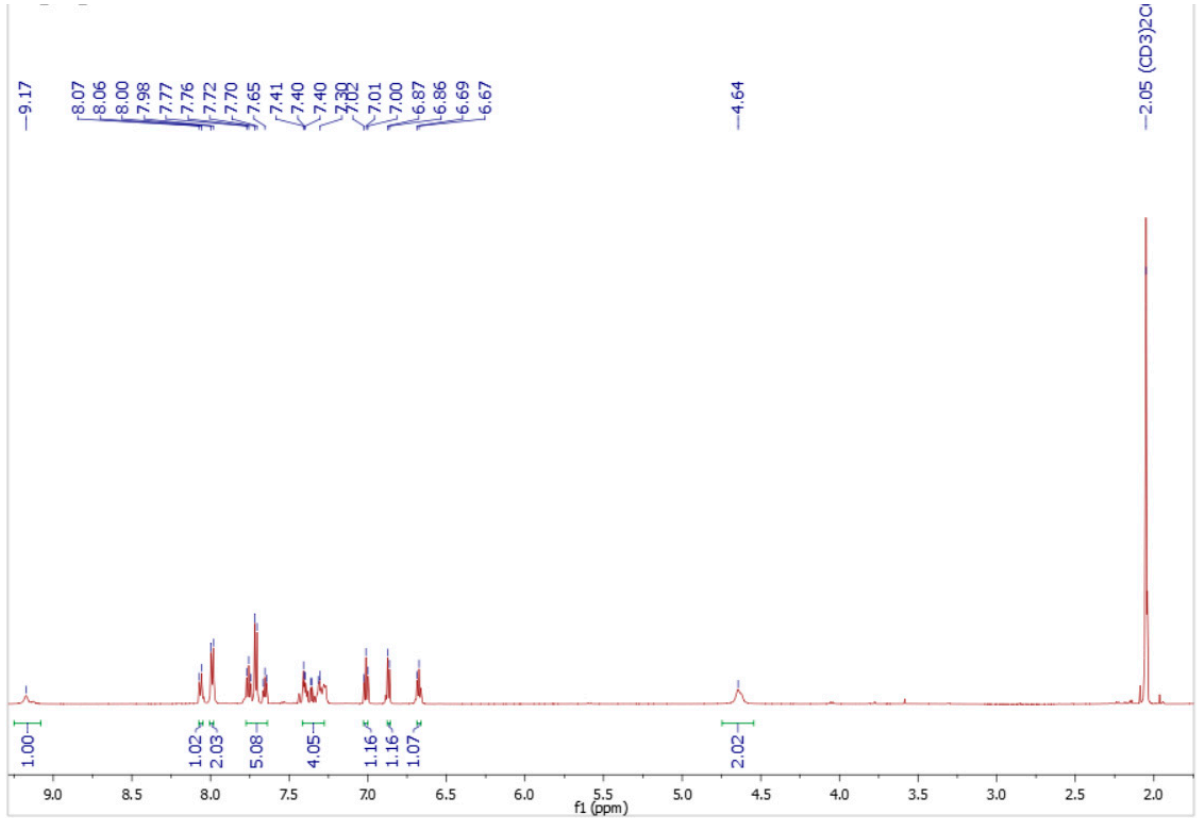

Figure S27: ^{13}C NMR spectra of compound (**7**) (150MHz, $(CD_3)_2CO$).

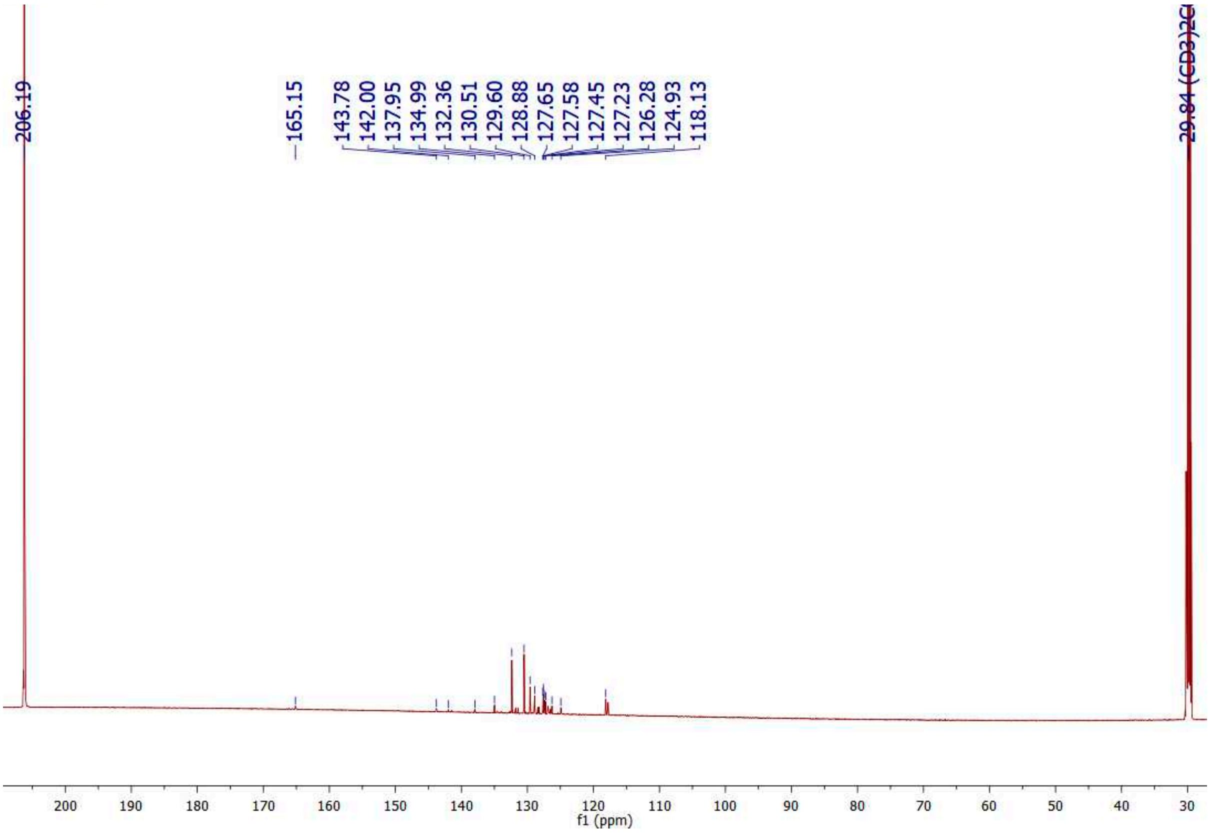

Figure S28: ^{1}H NMR spectra of compound (**20**) (600MHz, DMSO*d6).*

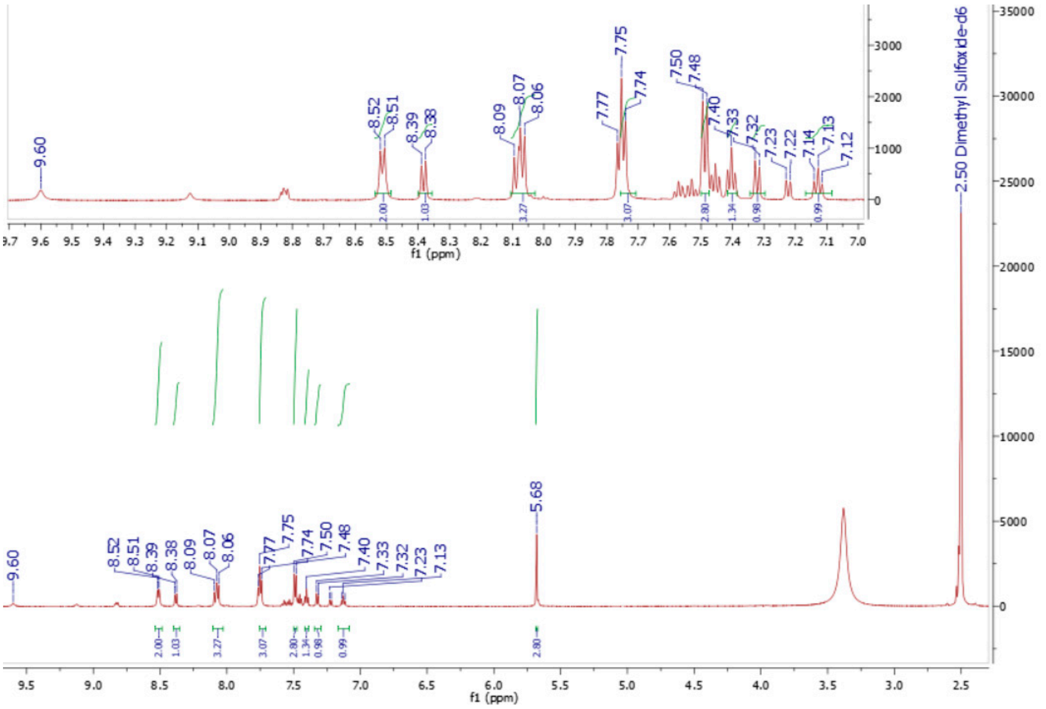

Figure S29: ^{13}C NMR spectra of compound (**20**) (150MHz, DMSO*d6).*

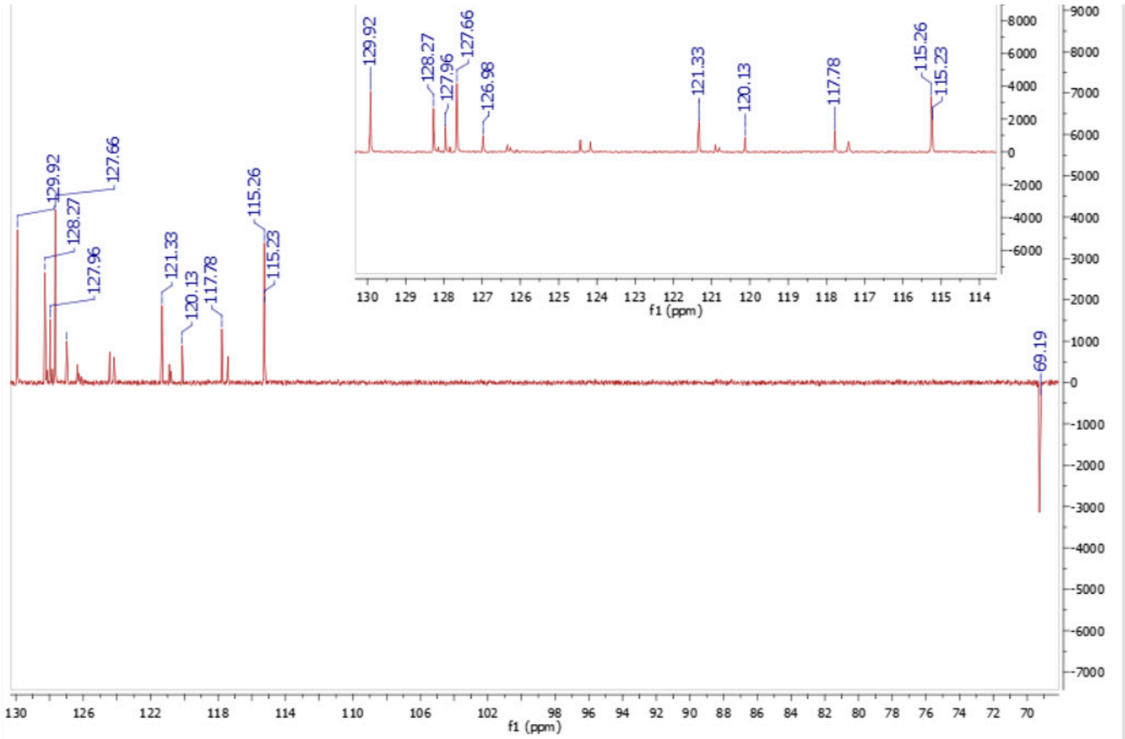

Figure S30: ^{1}H NMR spectra of compound (**21**) (600MHz, DMSO*d6).*

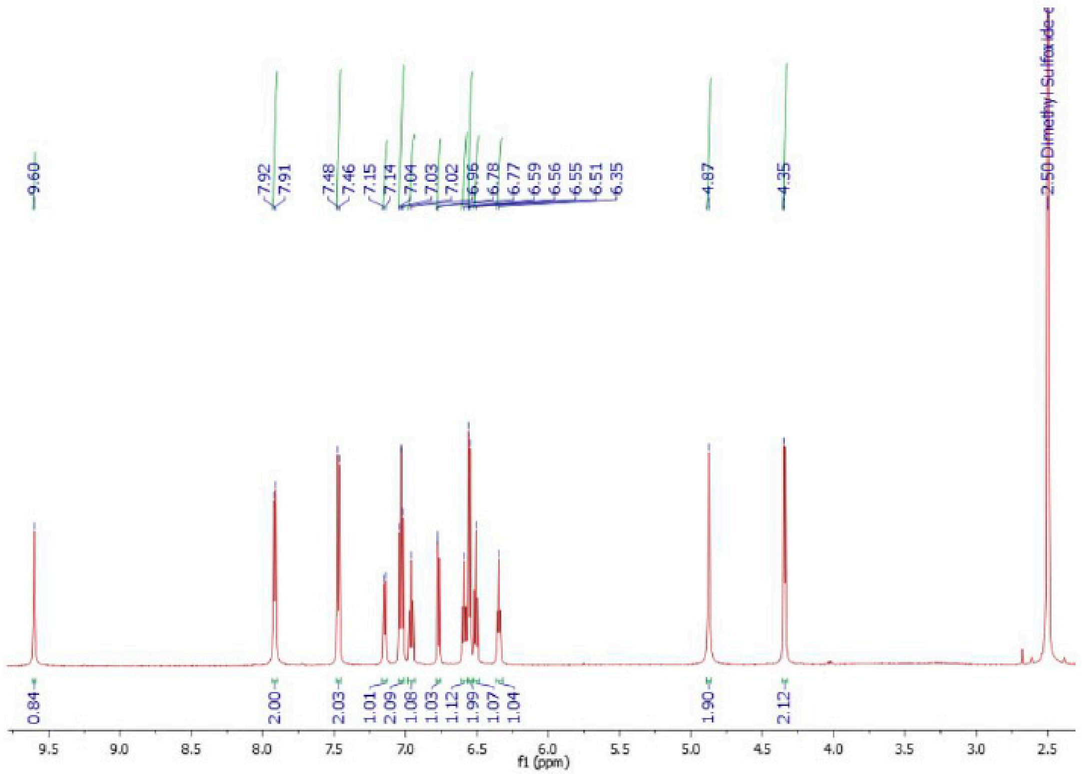

Figure S31: ^{13}C NMR spectra of compound (**21**) (150MHz, DMSO*d6).*

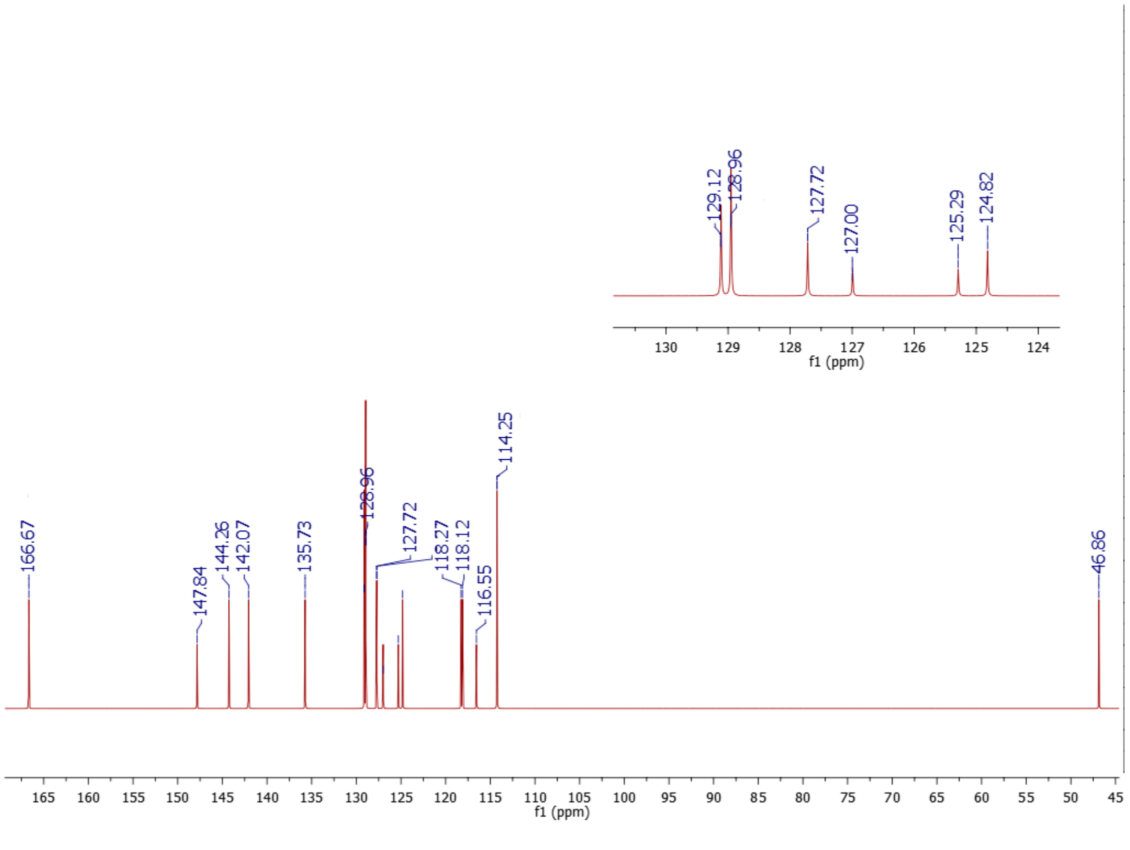

Figure S32: ^{1}H NMR spectra of compound (**22**) (600MHz, DMSO*d6).*

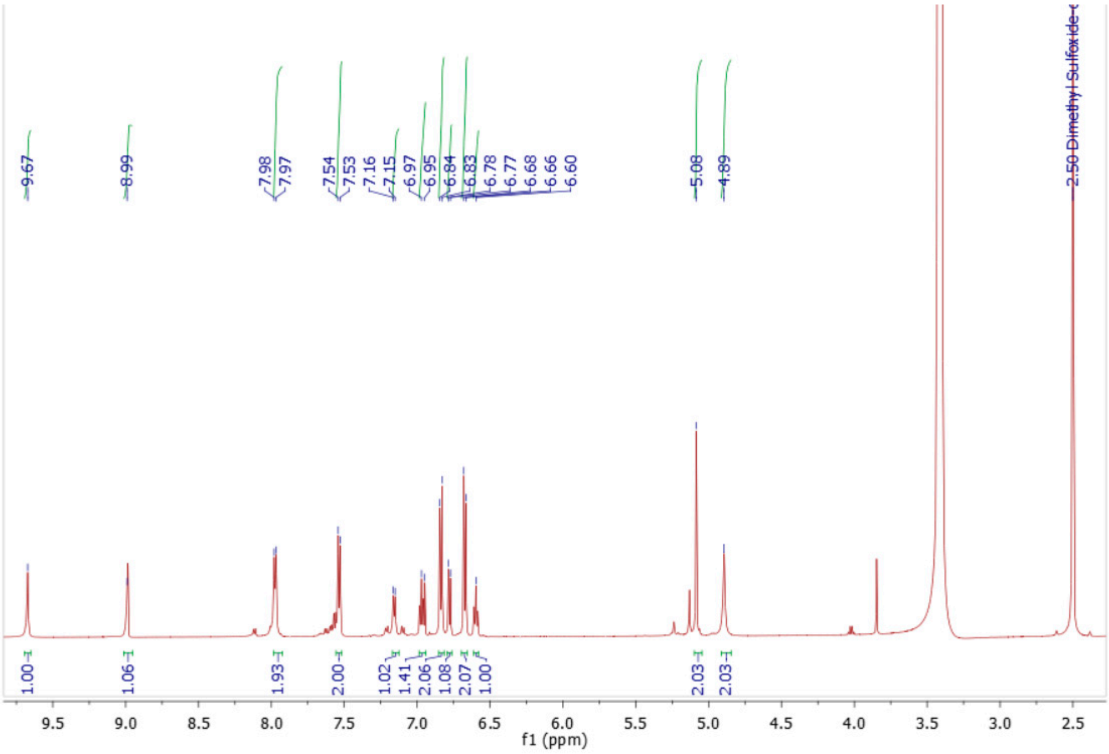

Figure S33: ^{13}C NMR spectra of compound (**22**) (150MHz, DMSO*d6).*

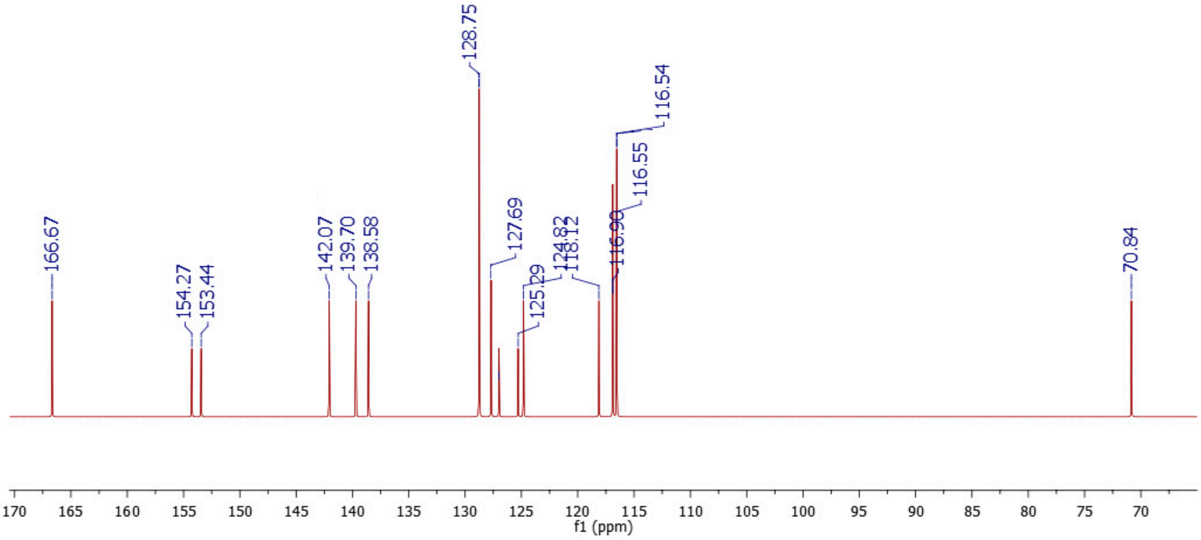

Figure S34: ^{1}H NMR spectra of compound (**23**) (600MHz, DMSO*d6).*

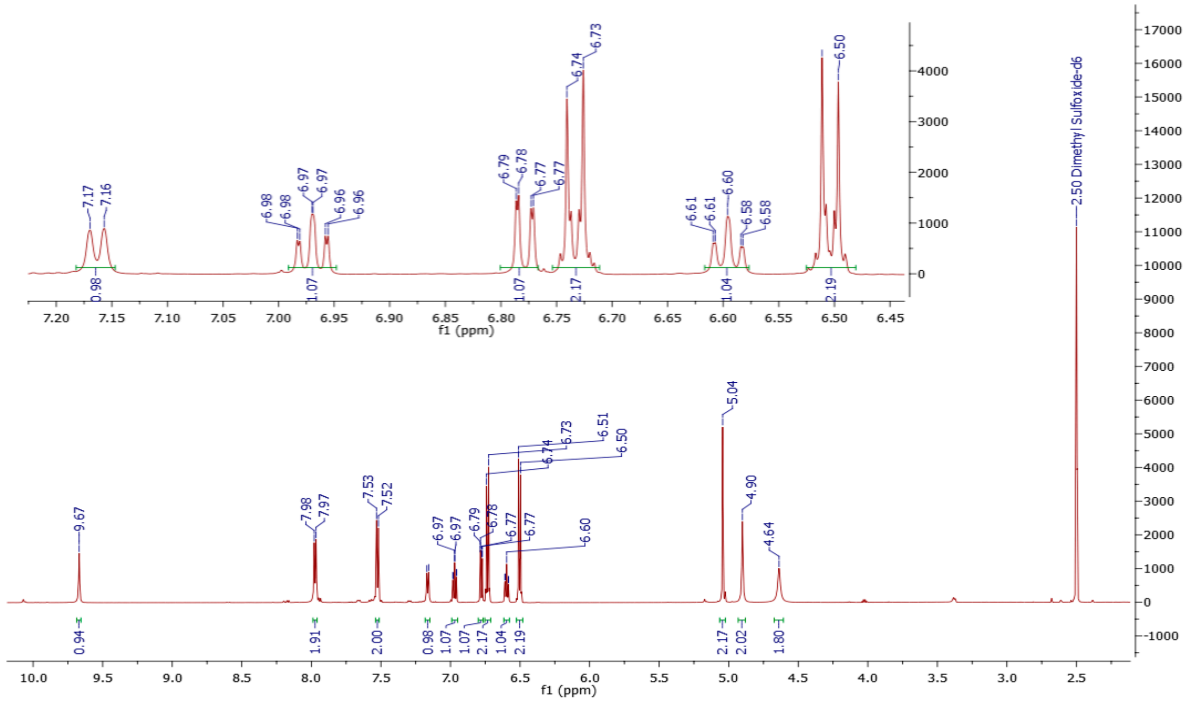

Figure S35: ^{13}C NMR spectra of compound (**23**) (150MHz, DMSO*d6).*

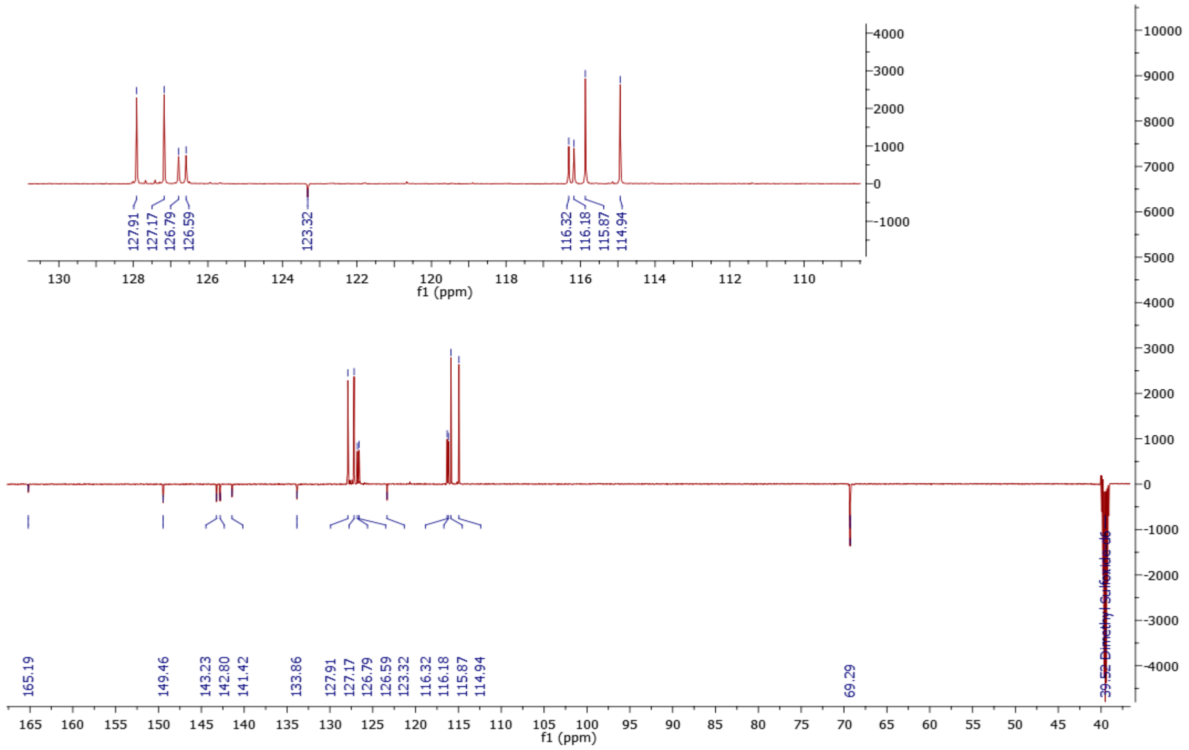

Figure S36: ^{1}H NMR spectra of compound (**24**) (300MHz, DMSO*d6).*

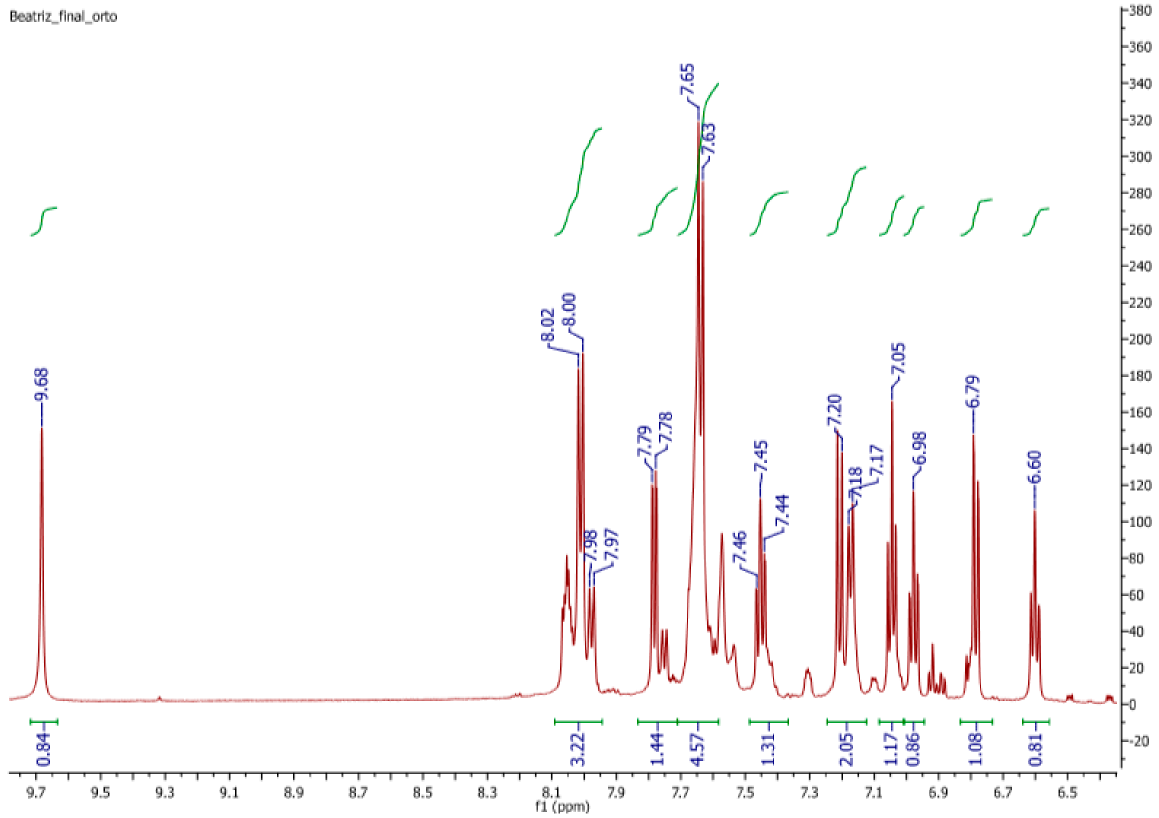

Figure S37: ^{13}C NMR spectra of compound (**24**) (75MHz, DMSO*d6).*

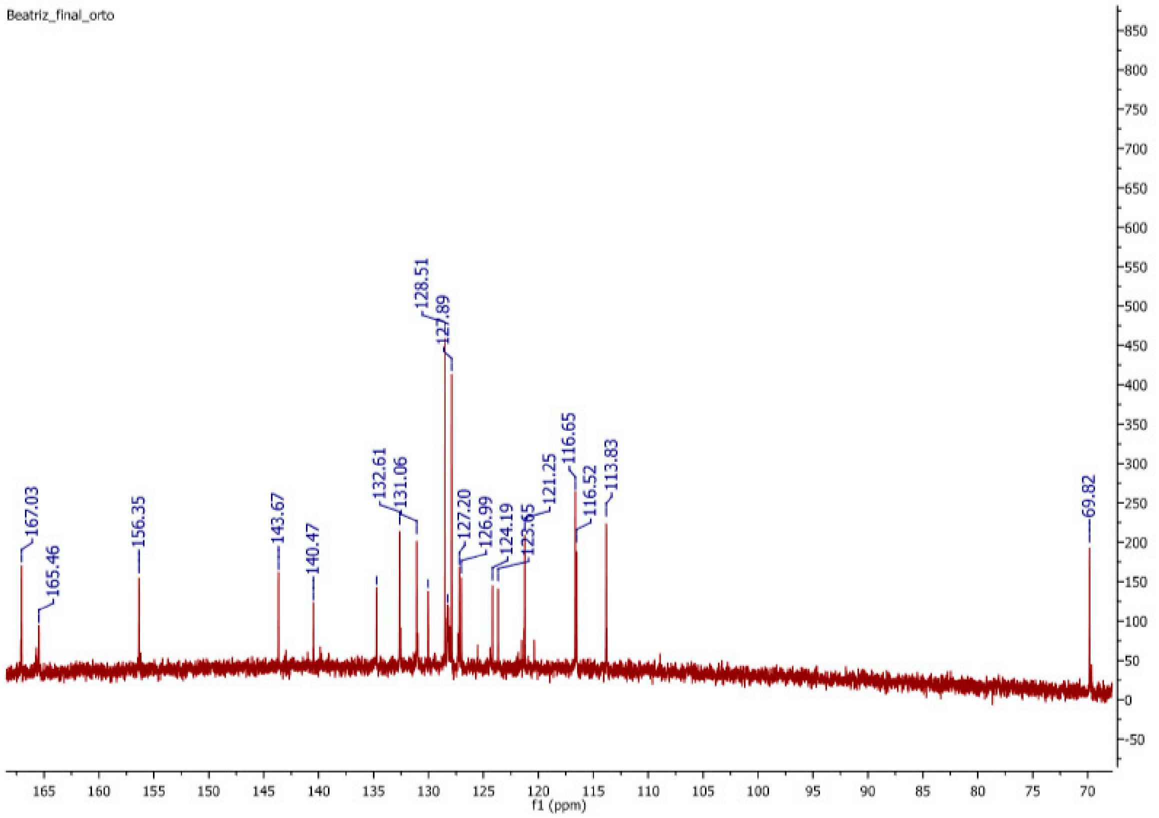

Figure S38: ^{1}H NMR spectra of compound (**25**) (600MHz, DMSO*d6).*

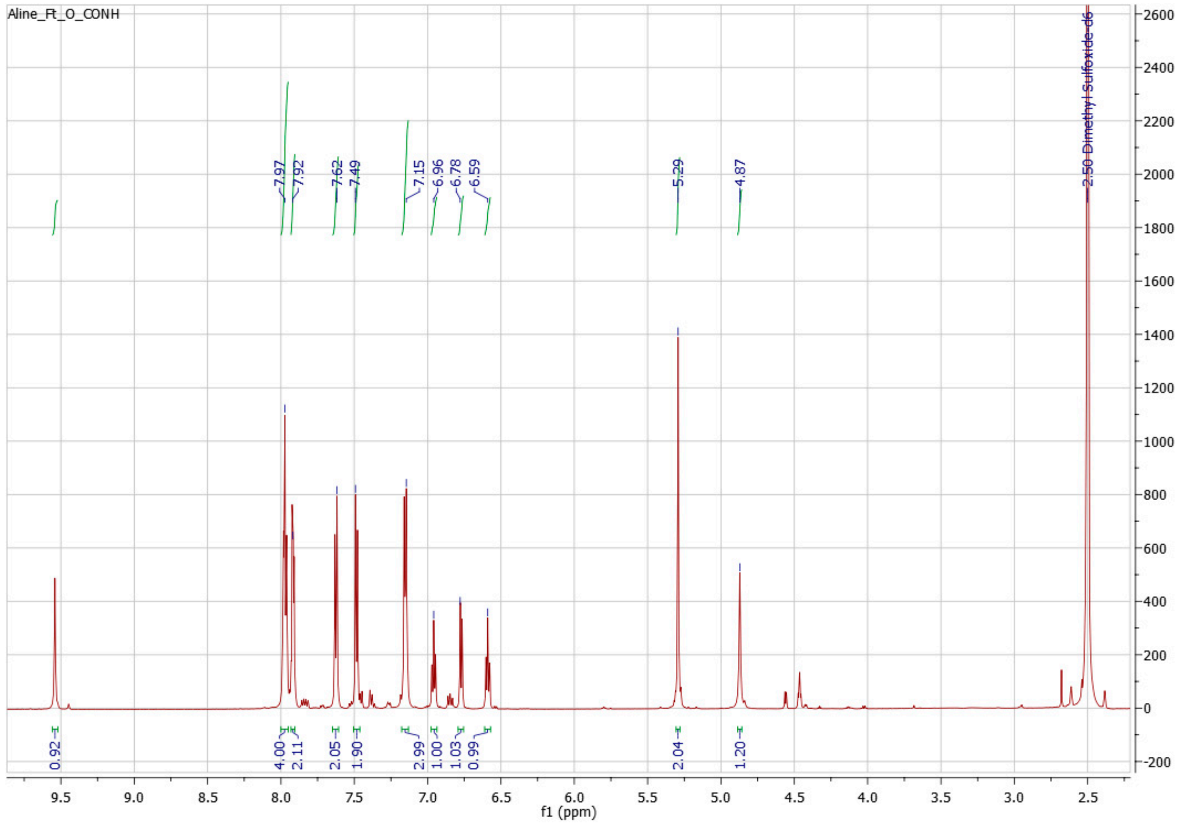

Figure S39: ^{13}C NMR spectra of compound (**25**) (150MHz, DMSO*d6).*

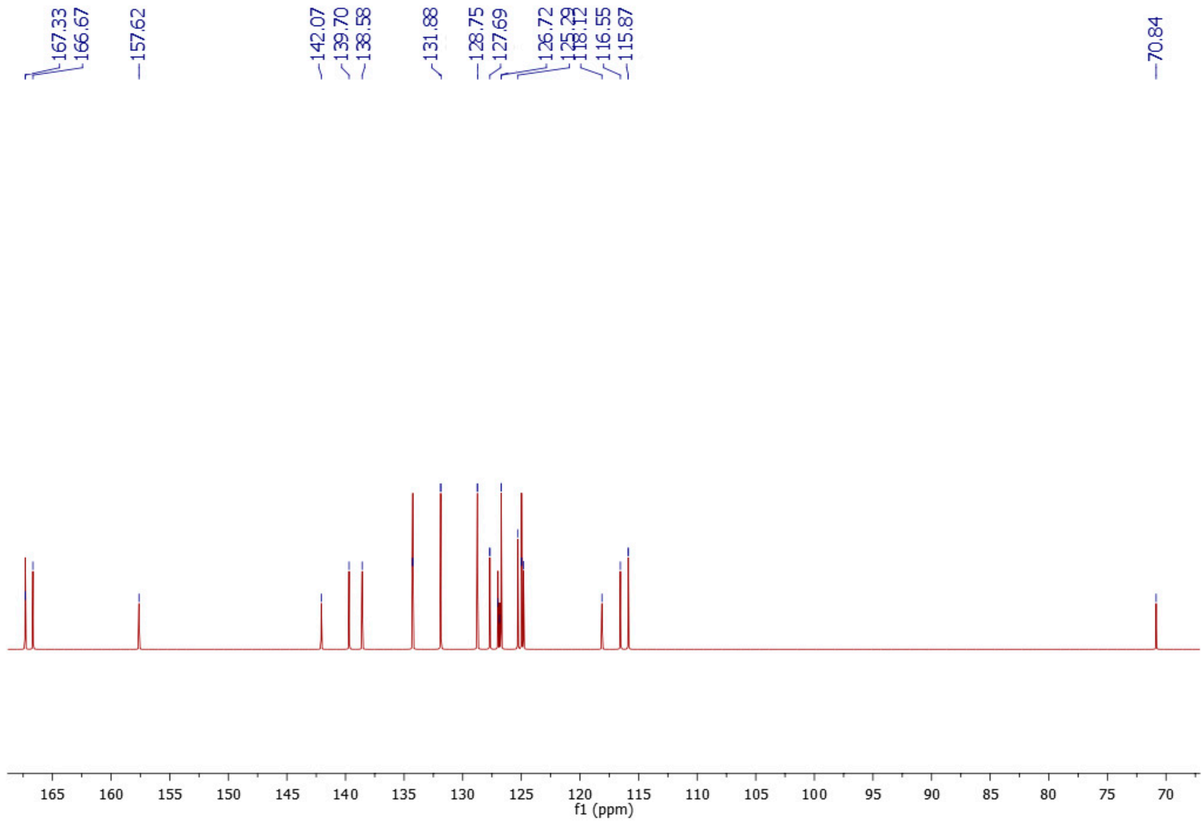

Figure S40: ^{1}H NMR spectra of compound (**26**) (600MHz, DMSO*d6).*

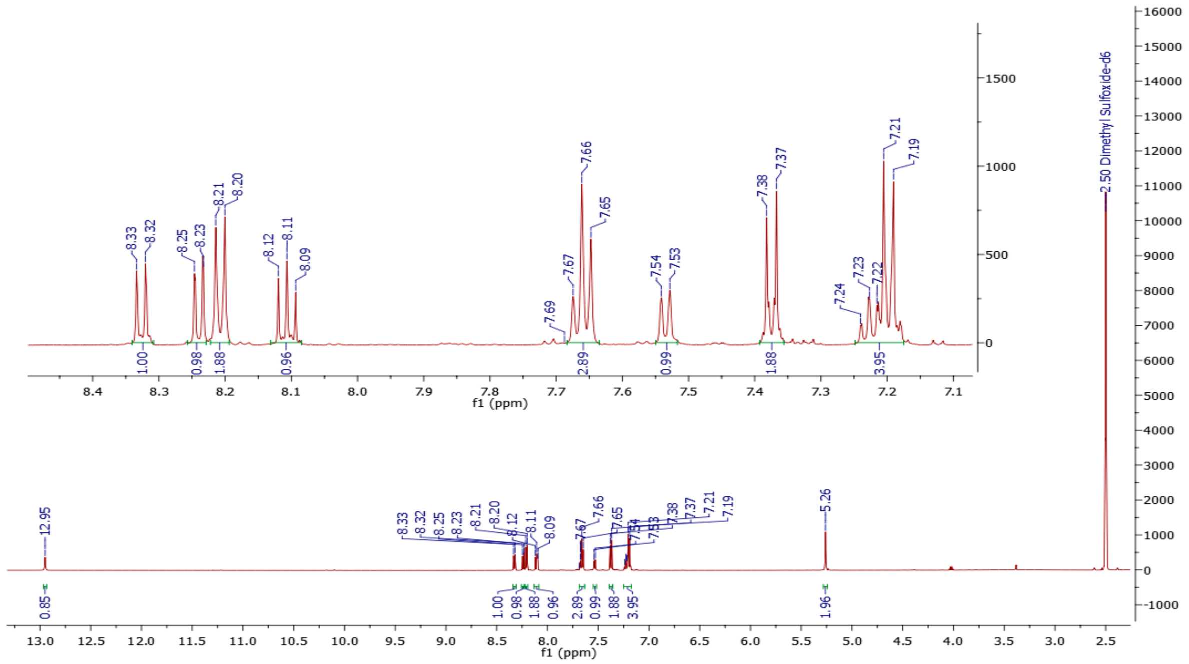

Figure S41: ^{13}C NMR spectra of compound (**26**) (150MHz, DMSO*d6).*

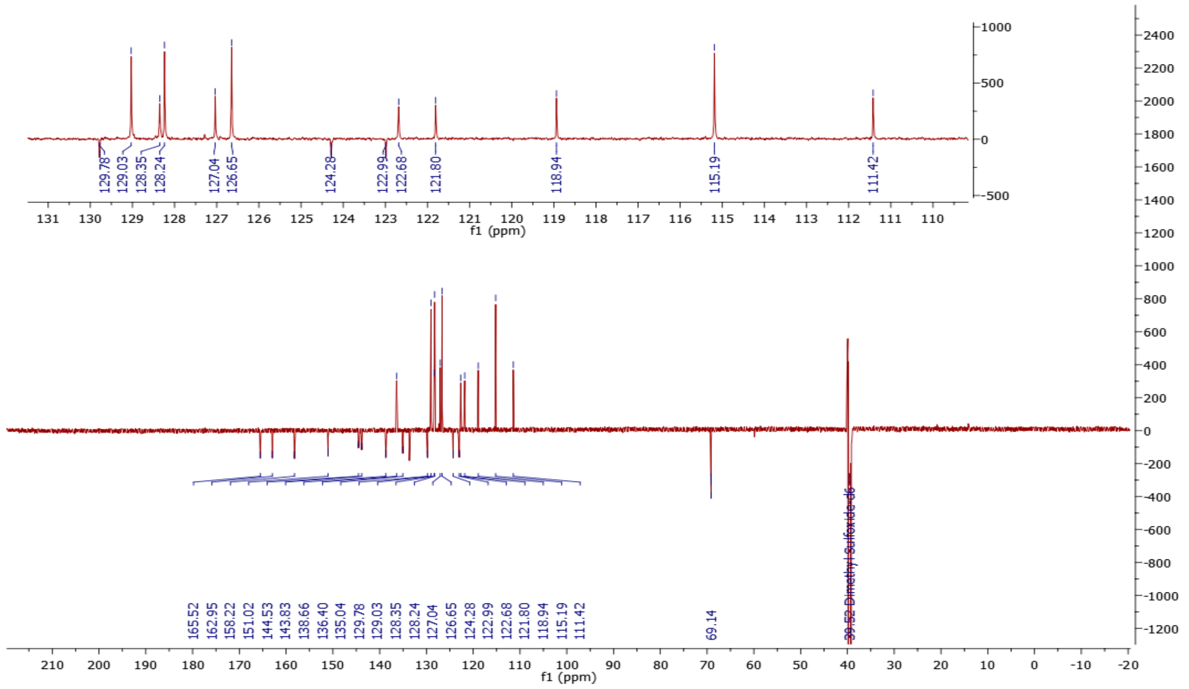

Supplement: Supplementary file 1 [file pharmaceuticals-15-01260-s001.zip › pharmaceuticals-1901579-Supplementary Materials.pdf]
